# Revision Total Hip Arthroplasty Using an Extensively Porous Coated Femoral Stem

**DOI:** 10.4055/cios.2009.1.2.105

**Published:** 2009-05-30

**Authors:** Kyoung Ho Moon, Joon Soon Kang, Sang Hyup Lee, Sae Rom Jung

**Affiliations:** Department of Orthopedic Surgery, College of Medicine, Inha University, Incheon, Korea.

**Keywords:** Revision total hip arthroplasty, Extensively porous coated femoral stem

## Abstract

**Background:**

To determine the benefit of an extensively porous coated femoral stem in patients receiving revision total hip arthroplasty.

**Methods:**

This study reviewed the results of 35 patients who received a revision total hip arthroplasty with extensively porous coated femoral stem between August, 1996, and December, 2002. The mean follow-up period was 77.5 months. The clinical and radiological results were evaluated by the Harris hip score and serial roentgenographic findings.

**Results:**

The preoperative and postoperative Harris hip score was 68.3 and 92.5, respectively. Radiographically, none of the acetabular components showed any evidence of migration, tilt, rotation, or shedding of metal particles. In addition, none of the femoral components showed evidence of subsidence, pedestal, or shedding of metal particles. Twenty-two hips had a mild stress shield and 2 hips had a moderate stress shield. The perioperative complications encountered were deep vein thrombosis (1 case), mild heterotopic ossification (4 cases), intraoperative periprosthetic fractures (1 case), and nonunion of the trochanteric osteotomy site (2 cases).

**Conclusions:**

Extensively porous coated femoral stems and acetabular components produce excellent clinical and radiological results in revision total hip arthroplasty.

The increase in total hip arthroplasty (THA) has led to higher incidence of revision arthroplasty caused by aseptic or septic loosening, osteolysis, periprosthetic fractures around the femoral stem, polyethylene wear, or recurrent dislocation. Various revision techniques have been introduced, such as cemented revision THA and femoral revision with a Wagner stem, modular stem, or proximally porous-coated stem.1) However, as bone loss is present in most cases where revision THA is required, it is not easy to obtain firm fixation of the femoral component in the proximal femur.^2^,3)

Fortunately, revision THA using an extensively porous-coated femoral stem allows fixation of the femoral component in a relatively well-preserved distal femoral shaft when there is a bone defect or severe loss in the femoral metaphysis.^4^,5) This study examined the efficacy of revision THA using an extensively porous-coated femoral stem in the study population consisting of 35 patients, who were followed up for more than 5 years.

## METHODS

**Fig. 1:**
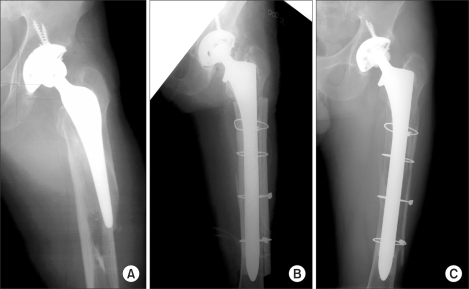
Radiographs show a 59 year-old man with periprosthetic fracture. (A) Preoperative radiograph reveals a periprosthetic fracture with Paprosky type III B femoral bone defect. (B) Revision total hip arthroplasty was performed with solution bowed stem and cortical strut allo-bone. (C) Five years after revision total hip arthroplasty, grafted cortical strut allo-bone had been incorporated .

From August 1996 to December 2002, 41 patients underwent revision THA using an extensively porous-coated cementless femoral stem at our hospital. Of them, 35 patients (35 hips) who were followed up for at least 60 months were enrolled in this study. There were 19 males and 16 females. The postoperative follow-up period averaged 77.5 months (range, 60 to 119 months). The causes of the primary hip arthroplasty were a fracture of the femoral neck in 14 cases, avascular necrosis in 9 cases, inflammatory arthritis in 8 cases, traumatic coxarthritis in 2 cases, and a congenital hip dislocation in 2 cases. As treatment, THA and bipolar endoprosthesis was performed in 25 and 10 patients, respectively. During these procedures, the acetabular cup and femoral stem were both cemented in 4 hips, only the acetabular cup was cemented in 1 hip, and only the femoral stem was cemented in 6 hips. The two components were cementless in 14 hips. A cementless femoral stem was implanted in 10 hips treated with bipolar endoprosthesis. The mean time from the primary surgery to revision was 85 months (range, 1 to 92 months). The causes of revision THA were aseptic loosening in 26 hips, septic loosening in 4 hips, a periprosthetic fracture around the femoral stem in 2 hips, polyethylene wear in 2 hips, and recurrent dislocation in 1 hip. Preoperatively, the hips were evaluated according to the Harris Hip score (HHS) and Paprosky's classification of bone stock deficiency.6) The first author of the study performed all procedures using the posterolateral approach. In all patients, neurolysis of the sciatic nerve was performed to maintain the sciatic nerve during surgery. In all cases, the presence of infection was examined by an intraoperative frozen biopsy of the joint capsule and Gram stain of the joint fluid. In 23 hips, an extended femoral trochanteric osteotomy was used to remove the femoral stem. The replacement of choice was an extensively porous-coated cementless femoral stem that was 0.5 mm larger than the last reamer used, and had a diaphyseal scratch fit of at least 5 cm. In cases where septic loosening was the cause of revision THA, prosthesis with antibiotic-loaded acrylic cement (PROSTALAC) was implanted after removing the prosthesis and more than 6 weeks of antibiotic treatment was carried out until the infection was proven to have healed by blood and clinical tests prior to surgery. The acetabular components used in the revision THAs are as follows: Duraloc (DePuy, Warsaw, IN, USA) 1200 series in 24 cases, 100 series in 4 cases, and Option cup (DePuy) in 5 cases. In 2 cases, only the femoral stems were replaced, one with a ceramic head and the other with a metal head. With regard to the femoral components, an anatomical medullary locking (AML) stem (DePuy), 170 mm in length, was implanted in 19 hips, and a solution bowed stem (DePuy), 205 mm in length, was used in 16 hips. The thickness of the stem was 10.5 mm in 1 hip, 12.0 mm in 1 hip, 13.5 mm in 10 hips, 15.0 mm in 10 hips, 16.5 mm in 5 hips, and 18.0 mm in 8 hips. According to the Paprosky's classification of acetabular bone defects, there were 6 type I, 8 type IIA, 7 type IIB, and 14 type IIIA hips, preoperatively. A morsellized allograft was used in the revision of 14 IIIA hips. According to the Paprosky's classification of femoral bone loss, there were 18 type I, 11 type II, 3 type IIIA, and 3 type IIIB hips preoperatively. A strut allograft was used in the revision of 3 type IIIB hips (Fig. 1).

The postoperative clinical and radiological assessments were performed at 6 weeks, 3 months, 6 months, and 12 months after surgery and on a yearly basis thereafter.

In the clinical assessment, the preoperative and postoperative HHS were compared. In the radiological assessment, the stability of the acetabular cup and the femoral stem was assessed by comparing the radiographs taken immediately after surgery with those at the last follow-up. To assess the stability of the acetabular cup, the level of cup migration, changes in tilting, rotation, radiolucent lines, shedding of metal particles were investigated according to the DeLee and Charnley zones. The cups were defined as being unstable if ≥ 2 mm migration, ≥ 5° changes in tilting, shedding of metal particles, and a continuous periacetabular radiolucent line were noted.7) The wear rate and volumetric wear of the liner in the acetabular component were measured using a Powerlook 2001XL flat bed imaging scanner (Umax Data System Inc., Taipei, Taiwan) and a computer assisted vector analysis program (Hip analysis program ver. 4.0, University of Chicago, Orthopaedic surgery, Chicago, USA) developed by Martell and Berdia8) The radiographs taken at 6 weeks postoperatively were used to reduce the errors resulting from creep. Except for the 5 hips where a ceramic liner was implanted during revision surgery, the remaining 30 hips with a polyethylene liner were included for the measurement of volumetric wear.

The femoral stem implantation was assessed by observing the subsidence, pedestal formation, and the presence of a periprosthetic radiolucent line according to the Gruen zones. A stem was defined as unstable, if the following were observed: there was more than 5 mm progressive subsidence, pedestal formation, a continuous radiolucent line around the stem, or the shedding of metal particles. Stress shielding was graded according to the criteria reported by Engh et al.9) and the results were analyzed according to gender, age, body mass index (BMI), and diameter of the femoral stem in an attempt to determine the risk factors. Heterotopic ossification, which is one of the complications of THA revision, was assessed using the Brooker classification.10) For Statistical analysis, a Chi square-test was performed using SPSS ver. 12.0 (SPSS Inc., Chicago, IL, USA). A *p* value < 0.05 was considered significant.

## RESULTS

In the clinical assessment, the mean HHS increased from 68.3 points (range, 52 to 82 points) preoperatively to 92.5 points (range, 75 to 99 points) at the last follow-up. In the radiological assessment, there was no migration, changes in tilt, rotation of the acetabular cup, or the shedding of metal particles in any of the 35 hips. In 3 hips, radiolucent lines were noted around the acetabular cup in DeLee and Charnley zone III but they were not continuous. The mean wear rate and mean volumetric wear was 0.190 mm/year and 100.109 mm^3^/year, respectively, at the last follow-up. There were no signs of subsidence, pedestal formation, and shedding of metal particles around the femoral stem. Radiolucent lines were observed in 6 cases, 5 in Gruen zone 1 and 1 in Gruen zone 4 but they were not continuous. The stability of the 35 femoral stems was assessed according to the criteria reported by Engh. Cortical bone ingrowth was observed in the distal portion of the porous coating in 26 hips, and stable fibrous ingrowth in 9 hips but none were considered unstable. There were 22 cases of mild stress shielding and 2 cases of moderate stress shielding. The relationships between various factors and stress shielding are as follows: stress shielding was found in 13 males and 11 females; in 10 patients aged < 60 years and 14 patients aged ≥ 60 years; in 14 patients whose BMI was < 25 kg/m^2^ and 10 patients whose MBI was ≥ 25 kg/m^2^; in 3 hips with a stem ≤ 13.5 mm in diameter and 21 hips with a stem ≥ 15.0 mm in diameter (Table 1). Deep vein thrombosis occurred as a complication in 1 hip, which was treated with a week of heparin therapy and 3 months of warfarin therapy. In addition, mild heterotopic ossification was observed in 4 hips and a fracture line was recognized in the femoral shaft during the insertion of the femoral stem in 1 hip. To achieve appropriate fixation, a control cable was used during hip surgery and bony union was confirmed in the follow-up period. There were 2 cases of nonunion of the greater trochanter following an extended trochanteric osteotomy but bony union could be obtained after a cancellous bone graft.

## DISCUSSION

The prevalence of THA has resulted in a higher incidence of THA use in younger patients. In addition, the number of revision THAs is also on the rise due to the longer life expectancy.11) A successful revision can be achieved by a femoral stem that ensures early stability and preservation of the normal biomechanism of the hip.12) For this reason, various types of femoral stems have been attempted. Cemented femoral stems and proximally porous-coated ones were used in the earlier days but the results were unsatisfactory.^11^,13) In contrast, extensively porous coated cementless femoral stems whose distal portions are designed for use in fixation to promote bone ingrowth and stability by being implanted in the relatively less impaired femoral diaphysis instead of the femoral metaphysis.^5^,6)

A cemented implant has been used in revision, if a patient is of advanced age, has severe osteoporosis, requires rapid weight bearing due to unilateral paralysis or systemic exacerbation, and in some cases if the medullary cavity of the femur is too wide or deformed to allow the use of a cementless stem. However, it is also known to produce high mechanical failure rates due to aseptic loosening resulting from decreased shear strength at the bone-cement interface.14) As proximally porous-coated femoral stems are usually press-fitted to the femoral metaphysis, their stability can be secured in the medullary cavity even when subsidence occurs soon after surgery. On the other hand, a severe bone deficiency in the proximal femur, which is common in most revisions, causes unsatisfactory results.4) In this study, 16.8% of 375 patients who had undergone revision using a proximally porous-coated femoral stem required additional revision during the 4.7 year follow-up. Moreland and Bernstein15) reported that only 2.9% of 175 hips failed after revision using an extensively porous-coated cementless femoral stem. Weeden and Paprosky5) also documented that a second revision was required in 6 out of 170 patients during a 14.2 year follow-up period. According to the 10 year follow-up study by Paprosky et al,16) 4.1% of the patients had poor outcomes, and 21% of failures were found in cases where the cortical bone-implant interface was less than 4 cm. Their results are consistent with other studies that focused on analyzing the relationship between the diameter of a femoral stem and the utmost stability in revision using an extensively porous-coated femoral stem. According to those studies, femoral bone loss requires at least 3 cm of diaphyseal contact with a stem for revision and excellent clinical results can be achieved when at least 4-6 cm of contact can be achieved.^16^,17) By maximizing the bone-implant interface and press-fitting the implant, an extensively porous-coated femoral stem can become effective in reducing femoral stem micromotion, promoting bone ingrowth and providing primary mechanical stability. Moreover, secondary stability can also be obtained through bone ingrowth in the distal portion, even when the bone quality of the proximal femur is bad and bone loss is severe.18) In the present study, bone ingrowth and obvious thickening of the cortical bone could be observed at the distal stem in 26 out of 35 hips and stable fibrous fixation was observed in 9 hips. Therefore, stable fixation of femoral implants could be confirmed in a mid-term follow-up study that lasted for more than 5 years.

Unfortunately, the use of an extensively porous-coated femoral stem can be problematic due to stress shielding both in primary and revision surgery. The more severe the osteoporosis and larger the diameter of a femoral stem are, the more evident the stress shielding becomes.4) In addition, severe stress shielding can result in poor clinical outcomes, even though mild cases can be understood as part of the bone remodeling process that does not cause clinical problems. In this study, mild stress shielding was observed in 22 hips except for 2 hips with moderate stress shielding. No statistical significance was found in the association between stress shielding and gender (*p* = 0.357), age (*p* = 0.227), and BMI (*p* = 0.147). However, femoral stems ≥ 15.0 mm or ≤ 13.5 mm in diameter were associated with stress shielding at a statistically significant level (*p* = 0.021).

In addition to the choice of femoral component, a precise assessment of bone stock loss and proper treatments are essential for improving the outcome of revision THA. In this study, synostosis and stable fixation of an implant could be achieved with a morsellized allograft in 14 IIIA type hips and with a strut allograft in 3 IIIB type hips classified according to the Paprosky's classification.

The complications of revision THA include systemic ones, such as pulmonary embolism, urinary infections, respiratory failure, and arrhythmia, surgical site infection, heterotopic ossification, nonunion or malunion of the greater trochanter, periprosthetic fracture around the femoral stem, dislocation of the hip, injuries to the nervous system or the blood system, and a limping gait. Weeden and Paprosky5) reported intraoperative fractures in 8% of their study population, hip dislocation in 7.1%, surgical site infections in 1.8%, and the requirement of a second revision in 4.1%. Engh et al.12) identified, in their study of 160 patients, infections in 0.6% and nonunion of the trochanteric osteotomy site in 10.4%, and found that revision was required in 10 patients. In this study, an anti-embolic stocking was used postoperatively for 6 weeks to prevent these complications, and radiation therapy was performed when heterotopic ossification was expected to occur. Bed rest was required for 6 postoperative weeks and rehabilitation therapy was begun immediately afterwards. Complications observed in patients enrolled in this study included 1 case of an intraoperative fracture (2.9%), 2 cases of nonunion of the trochanteric osteotomy site (5.7%), 4 cases of heterotopic ossification (11.4%), and 1 case of deep vein thrombosis (2.9%).

In revision THAs, extensively porous-coated cementless femoral stems provided firm and stable fixation of the acetabular and femoral components and produced good clinical results during the mid-term follow-up period. Therefore, extensively porous-coated cementless femoral stems are recommended as useful prostheses for revision THAs. However, their efficacy should be assessed using a longer-term study.

## Figures and Tables

**Table 1:**
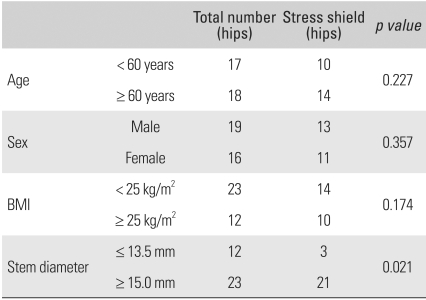
The Relationships of Variable Factors and Stress Shield
